# Effects of small-sided games and running-based high-intensity interval training on body composition and physical fitness in under-19 female soccer players

**DOI:** 10.1186/s13102-022-00516-z

**Published:** 2022-06-28

**Authors:** Sinan Nayıroğlu, Ali Kerim Yılmaz, Ana Filipa Silva, Rui Silva, Hadi Nobari, Filipe Manuel Clemente

**Affiliations:** 1 Faculty of Yaşar Doğu Sport Sciences, Ondokuz Mayıs University, Samsun, Turkey; 2 Escola Superior Desporto e Lazer, Instituto Politécnico de Viana do Castelo, Rua Escola Industrial e Comercial de Nun’Álvares, 4900-347 Viana do Castelo, Portugal; 3 Research Center in Sports Performance, Recreation, Innovation and Technology (SPRINT), 4960-320 Melgaço, Portugal; 4 Department of Exercise Physiology, Faculty of Educational Sciences and Psychology, University of Mohaghegh Ardabili, Ardabil, 5619911367 Iran; 5 Department of Motor Performance, Faculty of Physical Education and Mountain Sports, Transilvania University of Braşov, 500068 Brasov, Romania; 6 Department of Physiology, School of Sport Sciences, University of Extremadura, 10003 Cáceres, Spain; 7 Instituto de Telecomunicações, Delegação da Covilhã, 1049-001 Lisbon, Portugal

**Keywords:** Football, High-intensity interval training, Small sided games, Athletic performance, Human physical condition

## Abstract

**Background:**

The aim of this study was to compare the effects of small-sided games (SSGs) and running-based high-intensity interval training (HIIT) on the body composition and physical fitness of youth female soccer players.

**Methods:**

This study followed a randomized parallel study design. Twenty-four female soccer players (age: 18.63 ± 2.36 years) were randomly allocated to two training groups (SSG, n = 12; and HIIT, n = 12). The training intervention had a duration of eight weeks, consisting of three training sessions per week. Players were assessed twice (pre- and post-intervention) for anthropometrics, vertical (countermovement jumps, CMJ; and drop jumps, DJ) and horizontal jumping (single, triple and crossover hop), sprinting (10- and 30-m), change-of-direction (COD), COD deficit and final velocity at 30–15 Intermittent Fitness Test (V_IFT_). A covariance analysis (ANCOVA) was used to determine differences between the groups in the effect on post-intervention by controlling for covariates (pre-intervention). The within-group analysis (time) was performed using a paired *t*-test, while the between-group analysis per assessment moment was performed using an independent *t*-test.

**Results:**

The between-group analysis with ANCOVA revealed that there are no significant differences between the SSG and HIIT groups in the post-intervention for any outcome (*p* > 0.05). The within-group analysis revealed significant improvements in both the SSG and HIIT groups in CMJ (*p* < 0.05), single, triple and crossover hops (*p* < 0.05), RSI DJ 30-cm and RSI DJ 40-cm (*p* < 0.05), VIFT (*p* < 0.05) and COD (*p* < 0.05).

**Conclusions:**

SSG and HIIT are both effective for improving vertical and horizontal jumping ability, change-of-direction, and aerobic capacity status measured at a progressive and intermittent multistage test in youth soccer players.

## Background

Soccer is one of the most common and complex sports, and players need to possess high levels of physical, technical, and tactical determinants to be successful [1, 2]. Modern soccer is considered a much more demanding sport than it was in the past few years [3]. In previous studies, players’ HR was followed during matches [4–6]. In these studies, while the average HR of the players was around 86 bpm, the peak HR was around 98 bpm [4, 5]. In addition, during a 90-min match, female soccer players can cover 10–11 km^−1^ distance, 20% of which is covered at high intensities [4, 5, 7, 8].

The capability to perform and recover from high‐intensity activities is significant for soccer players, as soccer includes many explosive and intermittent actions [9, 10]. High-intensity interval training (HIIT) is one of the most effective training methods and is frequently used to improve the player’s cardiorespiratory and metabolic function [11–13]. HIIT interventions develop athletes’ resilience to high-intensity activities (i.e., activities involving greater than 85% of peak oxygen uptake in a shorter period than in traditional endurance training) [14, 15]. Additionally, it was previously shown that HIIT interventions can improve maximal oxygen uptake by approximately 5–11% and running economy by around 3–7% [16]. Moreover, given the analytical nature of HIIT interventions, coaches can better control the session intensity than in small-sided games (SSGs), as SSGs are associated with greater variations in session intensity [15, 17].

There are five different types of HIIT that coaches can implement to help athletes cope with soccer demands [18, 19]: (1) short HIIT, which consists of sub-maximal efforts less than 45 s; (2) long HIIT, which consists of sub-maximal efforts between 2–4 min; (3) repeated sprint training (RST), which consists of less than 10 s of repeated all-out short sprint series; (4) sprint interval training, which consists of more than 20–30 s of all-out sprinting with long rest periods; and (5) game-based training (e.g., generally, small-sided games [SSGs] used as a training intervention similar to long HIIT). These types of HIIT have different acute effects on the metabolic and neuromuscular systems [9, 20].

SSGs, which consist of smaller pitch sizes, different rules, and reduced player numbers compared to traditional soccer practices, are considered an enjoyable and effective training method to improve physical fitness and tactical skills in soccer [21–23]. Indeed, some SSGs’ intensities can be similar to those of soccer matches [22, 24]. SSGs are also considered a time-efficient training method, as they can develop technical skills, physical performance, and tactical awareness simultaneously [25]. All these advantages make SSGs a popular and ecological training method for the majority of soccer coaches [22].

Several studies have examined the effects of different SSGs and HIIT interventions in soccer [26–29]. According to these studies, there are no significant differences between SSG and HIIT in terms of perceived intensity. However, SSGs are more enjoyable than HIIT training methods [11]. Moreover, there are different SSGs formats (e.g., 3v3, 4v4, 6v6, among others) that produce diverse physiological responses [30]. However, the studies providing these results were conducted in male soccer players or in clinical populations, while no studies have been conducted on female soccer players contrasting the effects of both HIIT and SSG training interventions on under-19 female soccer players [11, 31].

The understanding of the effects of SSGs and HIIT on body composition and different physical fitness measures of female soccer players can give coaches and practitioners new insights into which type of intervention they should apply in their training programs, according to their individual needs. Hence, this study had two purposes: (1) to examine the effects of SSGs versus HIIT on under-19 female soccer players. Given that the overall SSGs and HIIT seem to present similar responses [11, 12], we hypothesized that SSGs methods are as effective as HIIT methods for improving the body composition and physical fitness of female soccer players.

## Materials and methods

### Study design

A randomized parallel matched-group design was used in the present study. All the players belonged to the same team and were divided into two different training intervention groups: (1) SSG (n = 12); and (2) HIIT (n = 12). A simple randomization system was used to divide the players into different teams. Two types of letters were prepared according to the number of players, and each player was requested to pick one. The participants picked the letters in alphabetical order and joined their groups.

The current study was conducted during the pre-season period of 2021–2022 in a team competing in the Turkey Second Women Football League. The study lasted 10 weeks and consisted of one week of tests (pre-testing), eight weeks of training intervention, and another week of tests (post-testing). Due to COVID-19 pandemic restrictions and lockdowns, no official matches were played in the Turkish Second Women League during the study period. Therefore, all participants participated only in training sessions and friendly matches.

### Subjects

G*Power software (version 3.1) was used to estimate the sample size. Using a partial effect size of 0.2, power of 0.8, *p* value of 0.05 (two groups and two measurements), and correlation of 0.5, we obtained a sample size of 12. Twenty-four female soccer players (age: 18.63 ± 2.36 years, height: 162.46 ± 4.94 cm, body mass: 54.18 ± 7.89 kg) participated in this study. All of the players were members of the same Turkish female soccer team. The players were assigned to two groups: the SSG group (n = 12) and the HIIT group (n = 12), see Table 1. The eligibility criteria were as follows: (1) subjects had no injuries, illness, or physical limitations during the study; (2) subjects completed at least 80% of the total sessions; and (3) subjects completed all of the test procedures (pre-post). The team trained with two different training methods, three days a week, in addition to the normal team training schedule. The team continued normal soccer training. Each training intervention lasted about 15–20 min, and each overall training session lasted about 75 min. The subjects did not play any official matches during the study period. Over the eight-week training period, one subject from the HIIT group, was removed from the study because of an injury. All players were notified of the research procedures, requirements, benefits, and risks, and they all signed a written informed consent obtained prior to the study. Also, this study’s design was confirmed by the Ondokuz Mayis University Ethics Committee (2021/362) and followed the ethical guidelines of the Declaration of Helsinki for the study of humans.

**Table 1:** Demographic information

Variables	SSG group	HIIT group	Total
Subjects (n)	12	12	24
Age (years)	18.8 ± 2.7	18.5 ± 2.1	18.6 ± 2.4
Soccer experience (years)	6.0 ± 2.5	5.3 ± 2.1	5.6 ± 2.3
Height (cm)	163.6 ± 5.2	161.3 ± 4.7	162.5 ± 4.9
Body mass (kg)	54.0 ± 7.3	54.8 ± 8.7	54.2 ± 7.9
BMI (kg/m^2^)	20.0 ± 2.2	21.1 ± 2.9	20.5 ± 2.6
Defenders (n)	4	5	9
Midfielders (n)	3	4	7
Attackers (n)	5	3	8
Adherence (%)	93.6	91.7	92.6

*BMI* body mass index, *SSG* small-sided games, *HIIT* high-intensity interval training

### Testing procedures

The assessments started at 4:00 p.m. each day, except for anthropometry measurements (10:00 a.m.). Players were requested to maintain their normal dietary intake on the assessment days. The players were introduced to the protocols of the tests and were organized into two different groups. Body mass, height, body mass index, and 30–15 intermittent tests were conducted on the first visit of the pre-season. On the second visit, the subjects were tested for 10- and 30-m linear sprinting and for 5–0–5 change-of-direction tests. On the third visit, the subjects completed three different hop tests (single leg, triple leg, crossover). During the last day of assessments, the subjects carried out countermovement jump and drop jump tests. The test assessments lasted four days in total, from Monday to Thursday. The subjects participated in a standard warm-up protocol consisting of 5 min of self-paced moderate running, five minutes of lower-limb dynamic stretching, and five minutes of reactive strength exercises focused on lower limbs before the tests.

### Anthropometry

We measured each athlete’s height by asking players to stand up in front of a wall with regular training shorts, a T-shirt, and no shoes. A body mass measuring instrument (Fakir, Germany) was used to assess the player’s weight. All these measures were taken two times to reduce the margin of error.

### Vertical jump

The countermovement jump (CMJ) with arms on hips and with a free arm test was used in the current study. The players were informed about the protocol of the tests and had two practice attempts to ensure their understanding of the tests. After the trials, the players performed the CMJ. Each subject had four official CMJ trials, two with their arms on their hips and two with their arms free, with a passive recovery of two minutes in between. Then, the best and average heights were calculated. Vertical jump data were collected using the Optojump system (Microgate, Italy). Jump height data were calculated automatically from flight time by Optojump. In the pre-test, CMJ coefficient of variation (CV) was 4.2% for the SSG group and 3.2% for the HIIT group; in the post-test, it was 3.8% CV for the SSG group and 2.7% CV for the HIIT group. The variability in the CMJ pre-test with free arms was 5.5% CV for the SSG group and 1.8% CV for the HIIT group; in the post-test, it was 3.9% CV for the SSG group and 4.2% for the HIIT group. For statistical analysis, the mean of each assessment moment (pre and post) was used.

### Horizontal jump

The single leg hop (SLH), triple leg hop (TLH), and crossover hop (CH) tests were used in this study to estimate the horizontal jump performance. The hop tests were performed on a course consisting of a 15-cm marking strip on the gym floor, which extended for 6 m. A tape measure was used to measure hop distance. Before the tests, all players were allowed to perform one trial for practice. The players also performed two trials for each hop test and each leg. The jump distance was taken after landing, measured from the nearest contact point (back of the heels). The best result was recorded for further data treatment. There was a 30-s passive resting period for different leg trials in the same test. However, the resting time between different hop tests was two minutes. The symmetry angle was calculated for each hop test. The symmetry angle calculation was based on the following formula: (45 − arctan (non-dominant leg/dominant leg))/90 × 100. The hop test CV values of SSG group were 6.5% (pre) and 7.0% (post) for SLH right, 8.8% (pre) and 8.0% (post) for SLH left, 10.7% (pre) and 9.1% (post) for TLH right, 9.6% (pre) and 11.1% (post) for TLH left, 7.4% (pre) and 9.5% (post) for CH right, and 9.5% (pre) and 10.0 (post) for CH left. The CV values of the HIIT group were 10.5% (pre) and 8.0% (post) for SLH right, 8.6% (pre) and 7.1% (post) for SLH left, 9.4% (pre) and 6.0 (post) for TLH right, 10.1% (pre) and 8.6% (post) for TLH left, 9.1% (pre) and 6.7% (post) for CH right, and 9.2% (pre) and 8.5% (post) for CH left. For statistical analysis, the mean of each assessment moment (pre and post) was used.

### Reactive strength index

The drop jump (DJ) test was conducted using different box heights (20 cm, 30 cm, and 40 cm) to measure the reactive strength index (RSI) [32]. All jumps were assessed using an Optojump photoelectric cells system (Microgate, Bolzano, Italy) connected to a portable computer with its respective software (Optojump software, version 1.10.19). The players were familiarized with the test before official trials, and 30 s of passive rest was given to the players between each trial. The players were requested to place their hands on their hips and were not allowed to use their arms. The players stepped off from the box with the preferred leg. They landed with two legs on the ground and jumped back as high as possible. Players were asked to minimize the contact time and maximize jump height. They performed three drop jump heights in an incremented height order (20 cm, 30 cm, and 40 cm). The RSI CV percentage of the SSG group in the pre-test was 20.0% on the 20-cm box, 17.3% on the 30-cm box, and 5.8% on the 40-cm box. In the post-test, it was 11.6% on the 20-cm box, 19.0% on the 30-cm box, and 20.1% on the 40-cm box. The pre-test RSI CV percentage of the HIIT group was 25.8% on the 20-cm box, 24.0% on the 30-cm box, and 15.8% on the 40-cm box. In the post-test, it was 20.1% on the 20-cm box, 19.8% on the 30-cm box, and 20.2% on the 40-cm box. For statistical analysis, the mean of each assessment moment (pre and post) was used.

### Sprint test

10-m and 30-m linear sprinting tests were used in this study. Two pairs of photocells (Witty Gate, Microgate, Italy) were used. The starting point was marked 30 cm behind the first pair of photocells. Players performed all trials using their preferred foot. They performed two trials, with two minutes of passive resting time given for each sprint [33]. The 10-m pre-test CV percentage was 1.2% in the SSG group and 1.0% in the HIIT group. In the post-test, it was 1.3% in the SSG group and 1.6% in the HIIT group. On the other hand, the SSG group had 0.7% pre-test and 0.9% post-test CV percentages for the 30-m linear sprinting test. Also, the HIIT group had 0.7% pre-test t and 0.8% post-test CV percentages for the 30-m test. For statistical analysis, the mean of each assessment moment (pre and post) was used.

### Change of direction

The players performed the 5–0–5 test so that we could measure change-of-direction (COD) time and deficit. Cones were placed at 0, 5, and 15 m, and the photocells (Witty Gate, Microgate, Italy) were placed at 5 m. The players started to accelerate from the 15-m point, and photocells were launched when they passed the 5-m point. When the players reached the 0-m point, they made a 180-degree turn and ran back to the 5-m point to finish the test. Before the test, the subjects had two practice trials to become familiar with the test [34]. Also, they were allowed to choose their preferred leg for turning during the COD task. Likewise, they were asked to put their preferred leg in front at the starting position. Each player performed two trials. The COD deficit was calculated by using the following formula: COD time minus 10-m linear speed time. The COD CV percentage of the SSG group was 1.8% in the pre-test, and 1.6% in the post-test. For the HIIT group, the CV percentage was 1.5% in the pre-test, and 1.4% in the post-test. For statistical analysis, the mean of each assessment moment (pre and post) was used.

### Aerobic fitness status

The 30–15 Intermittent Fitness Test (30–15 IFT) was carried out to analyze the aerobic fitness status of the players. The original 40-m version of the 30–15 IFT was used in this study. It includes 30-s shuttle runs with 15-s passive recovery periods. The starting speed was 8 km/h, and the speed was increased by 0.5 km/h at each level [13, 35]. The test ended when a player reached exhaustion or failed three times to reach the 3-m zone. The final velocity (km/h) of the last completed run was recorded for each player as the final outcome of the test. The CV percentage for the SSG group was 10.4% in the pre-test, and 9.1% in the post-test. For the HIIT group, it was 9.2% in the pre-test, and 8.1% in the post-test.

### Training intervention

The training intervention occurred three days a week, on Monday, Wednesday, and Friday. The training sessions of the team started at 4:00 p.m. During the training period, the average temperature was approximately 18 °C. After a standard warm-up protocol consisting of 5 min of self-paced moderate running, five minutes of lower-limb dynamic stretching, and five minutes of reactive strength exercises focused on the lower limbs, the players were separated into the groups and started the training protocol (Table 2).

**Table 2:** Training intervention details

Period of time	Group SSG	Group HIIT
Week 1 and 3 (session 1–3,7–9)	SSG format: 3v3 Pitch size: 18 × 30 m Coach encouragement: Yes No goals (aim: possession of the ball) No offside Sets: 2 Repetitions: 2 Work: 90 s Rest: 90 s Rest between sets: 4 min %65V_IFT_	Sets: 2 Repetitions: 6 Work: 15 s Rest:15 s Rest between sets: 4 min %65V_IFT_ Work intensity: 90–95%V_IFT_ Rest intensity: 0%V_IFT_
Week 2 and 4 (session 4–6,10–12)	SSG format: 2v2 Pitch size: 12 × 24 m Coach encouragement: Yes No goals (aim: possession of the ball) No offside Sets: 2 Repetitions: 2 Work: 90 s Rest: 90 s Rest between sets: 4 min %65V_IFT_	Sets: 2 Repetitions: 6 Work: 15 s Rest:15 s Rest between sets: 4 min %65V_IFT_ Work intensity: 90–95%V_IFT_ Rest intensity: 0%V_IFT_
Week 5 and 7 (session 13–15,19–21)	SSG format: 3v3 Pitch size: 18 × 30 m Coach encouragement: Yes No goals (aim: possession of the ball) No offside Sets: 3 Repetitions: 2 Work: 90 s Rest: 90 s Rest between sets: 4 min %65V_IFT_	Sets: 3 Repetitions: 6 Work: 15 s Rest:15 s Rest between sets: 4 min %65V_IFT_ Work intensity: 90–95%V_IFT_ Rest intensity: 0%V_IFT_
Week 6 and 8 (session 16–18, 22–24)	SSG format: 2v2 Pitch size: 12 × 24 m Coach encouragement: Yes No goals (aim: possession of the ball) No offside Sets: 3 Repetitions: 2 Work: 90 s Rest: 90 s Rest between sets: 4 min %65V_IFT_	Sets: 3 Repetitions: 6 Work: 15 s Rest:15 s Rest between sets: 4 min %65V_IFT_ Work intensity: 90–95%V_IFT_ Rest intensity: 0%V_IFT_

*SSG* small sided games, *HIIT* high intensity interval training, *V*_*IFT*_ final velocity at 30-15IFT

### Training intensity during the intervention

The monitoring of training intensity was determined with the rating of perceived exertion (RPE) by using the CR-10 Borg’s scale [36] in all training interventions. The players were familiarized with the scale before the interventions. The players were asked to individually score the perceived level of effort performed during the intervention. The question, “How was your workout?” was asked to the players and asked to evaluate the intensity of the intervention. The answers were recorded on a sheet after the question was answered.

### Statistical procedures

Preliminary inspection of normality and homogeneity of the sample was performed using Shapiro–Wilk test and Levene’s test, respectively. For the group of outcomes, normality was assumed since *p* > 0.05. Similarly, homogeneity was assumed since *p* > 0.05. Considering those assumptions, a covariance analysis (ANCOVA) was executed to detect differences between-groups by controlling for covariates (pre-intervention) [37]. Differences were reported in the format of *p* value and partial eta squared ($\eta_{p}^{2}$) from the ANCOVA output. Within group variations were tested using t-paired sample test, while between-group differences for baseline and post-intervention moments were tested using independent *t*-test. Magnitude of differences were tested using the standardized effect size of Cohen, with the equation ((mean post − mean pre)/(pool standard deviation)) [38]. The magnitude of differences was interpreted using the following thresholds [38]: [0.0;0.2], trivial; [0.2;0.5], small; [0.5;0.8], medium; > 0.8, large. SPSS Statistics software (version 24, IBM Corporation, Armonk, NY, USA) was used for the analysis.

## Results

Descriptive statistics of pre- and post-test anthropometric values, within-group and between-group analysis can be found in Table 3. Significant differences were detected between pre- and post-test for the case of body mass (*p* = 0.002) and BMI (0.003) in SSG group but no significant differences for height (*p* > 0.05). No significant pre- and post-test differences for HIIT group (*p* > 0.05). No significant differences (*p* > 0.05) were found between SSG and HIIT either for baseline or post-intervention for the outcomes presented in Table 3.

**Table 3:** Descriptive statistics (mean and standard deviation) of anthropometrics pre- and post SSG and HIIT interventions

Variables	Pre (m ± SD)				Post (m ± SD)			
	SSG	HIIT	%95 CI	*p* (indepent sample *t* test)	SSG	HIIT	%95 CI	ANCOVA, *p* ($\eta_{p}^{2}$)
Age	18.8 ± 2.7	18.5 ± 2.1			19.0 ± 2.8	18.7 ± 2.2		
Body mass (kg)	53.5 ± 7.3	54.8 ± 8.7	− 8.12 to 5.49	0.692	54.5 ± 7.4	54.5 ± 8.0	− 0.196 to 2.74	0.086 (0.134)
Height (cm)	163.6 ± 5.2	161.3 ± 4.7	− 1.94 to 6.44	0.277	163.8 ± 5.2	161.5 ± 4.6	− 0.263 to 0.483	0.546 (0.018)
BMI (kg/m^2^)	20.0 ± 2.2	21.1 ± 2.9	− 3.34 to 1.05	0.292	20.3 ± 2.2	20.8 ± 2.7	− 0.136 to 1.100	0.119 (0.111)

*BMI* body mass index, *SSG* small sided games, *HIIT* high intensity interval training, *ANCOVA* analysis of covariance, *CI* confidence interval

Descriptive statistics of pre- and post-test jumping values, within-group and between-group analysis can be found in Table 4. Significant differences between pre- and post-test were found for the case of CMJ (*p* < 0.05), CMJ free arm (*p* < 0.05), Single Hop Right (*p* < 0.05), Single Hop Left (*p* < 0.05), Triple Hop Right (*p* < 0.05), Triple Hop Left (*p* < 0.05), Crossover Hop Right (*p* < 0.05) and Crossover Hop Left (*p* < 0.05) in both the SSG and HIIT groups. In addition, significant differences in HIIT group for Triple Hop Symmetry Angle (*p* = 0.006), RSI DJ 30-cm (*p* = 0.019) and RSI DJ 40-cm (*p* = 0.026). No significant differences pre-to-post in SSG for Single Hop Symmetry Angle (*p* = 0.742), Triple Hop Symmetry Angle (*p* = 0.133), Crossover Symmetry Angle (*p* = 0.143), RSI DJ 20-cm (*p* = 0.597), RSI DJ 30-cm (*p* = 0.096) and RSI DJ 40-cm (*p* = 0.467). No significant differences pre-to-post in HIIT group for the Single Hop Symmetry Angle (*p* = 0.944), Crossover Symmetry Angle (*p* = 0.285) and RSI DJ 20-cm (*p* = 0.051).

**Table 4:** Descriptive statistics (mean and standard deviation) of jumping performance, sprinting, change-of-direction and aerobic fitness status pre- and post SSG and HIIT interventions

Variables	Pre (m ± SD)				Post (m ± SD)			
	SSG	HIIT	%95 CI	*p* (indepent sample *t* test)	SSG	HIIT	%95 CI	ANCOVA, *p* ($\eta_{p}^{2}$)
CMJ (cm)	23.9 ± 2.6	21.4 ± 1.9	0.55 to 4.40	0.014*	26.7 ± 3.2	23.6 ± 2.9	− 1.20 to 4.07	0.271 (0.057)
CMJ free arm (cm)	28.7 ± 4.00	25.5 ± 2.5	0.42 to 6.06	0.026*	31.6 ± 4.1	28.1 ± 3.5	− 1.74 to 3.77	0.453 (0.027)
Single hop right (cm)	156.2 ± 10.2	144.0 ± 15.1	1.32 to 23.2	0.030*	163.2 ± 11.4	153.7 ± 12.3	− 6.54 to 8.95	0.750 (0.005)
Single hop left (cm)	150.5 ± 13.2	142.2 ± 12.2	− 2.53 to 19.03	0.127	161.4 ± 12.8	152.5 ± 10.9	− 4.44 to 13.02	0.318 (0.047)
Triple hop right (cm)	490.6 ± 52.4	453.1 ± 42.7	− 2.93 to 77.94	0.068	515.7 ± 46.8	479.7 ± 29.0	− 11.40 to 39.59	0.263 (0.059)
Triple hop left (cm)	484.3 ± 46.5	441.2 ± 44.5	4.53 to 81.64	0.030*	510.0 ± 56.7	483.2 ± 41.6	− 39.11 to 14.32	0.346 (0.042)
Crossover hop right (cm)	456.6 ± 33.8	416.9 ± 38.0	9.25 to 70.08	0.013*	484.9 ± 46.0	450.1 ± 30.3	− 22.51 to 38.62	0.589 (0.014)
Crossover hop left (cm)	434.4 ± 41.1	415.8 ± 38.2	− 15.00 to 52.16	0.263	486.7 ± 48.9	452.7 ± 38.4	− 5.50 to 40.01	0.130 (0.106)
Single hop symmetry angle	2.1 ± 1.8	2.2 ± 1.9	− 1.71 to 1.47	0.874	1.9 ± 1.2	2.2 ± 1.8	− 1.66 to 0.980	0.598 (0.013)
Triple hop symmetry angle	3.0 ± 2.6	2.6 ± 1.2	− 1.55 to 1.91	0.834	2.0 ± 1.5	1.7 ± 1.4	− 0.81 to 1.24	0.671 (0.009)
Crossover hop Symmetry angle	2.7 ± 2.4	2.0 ± 1.0	− 0.82 to 2.32	0.332	1.6 ± 1.2	1.5 ± 1.2	− 1.06 to 0.98	0.938 (0.000)
RSI DJ 20 cm	1.83 ± 0.36	1.50 ± 0.39	0.01 to 0.65	0.045*	1.87 ± 0.22	1.7 ± 0.3	− 0.187 to 0.239	0.802 (0.003)
RSI DJ 30 cm	1.94 ± 0.33	1.65 ± 0.40	− 0.02 to 0.60	0.066	2.17 ± 0.41	1.88 ± 0.37	− 0.193 to 0.434	0.433 (0.030)
RSI DJ 40 cm	1.96 ± 0.31	1.68 ± 0.36	− 0.00 to 0.56	0.051	2.06 ± 0.41	1.85 ± 0.37	− 0.287 to 0.33	0.880 (0.001)
10 m sprint test (sec)	2.02 ± 0.08	2.15 ± 0.09	− 0.20 to − 0.60	0.001*	2.01 ± 0.08	2.08 ± 0.12	− 0.047 to 0.119	0.271 (0.057)
30 m sprint test (sec)	4.97 ± 0.19	5.27 ± 0.23	− 0.50 to − 0.12	0.002*	5.00 ± 0.17	5.20 ± 0.18	− 0.059 to 0.110	0.453 (0.027)
V_IFT_ (km/h^−1^)	15.2 ± 1.6	14.9 ± 1.4	− 0.93 to 1.59	0.589	16.5 ± 1.5	16.5 ± 1.3	− 0.856 to 0.390	0.750 (0.005)
COD time (sec)	2.72 ± 0.11	2.87 ± 0.10	− 0.24 to − 0.06	0.002*	2.59 ± 0.09	2.77 ± 0.11	− 0.192 to − 0.017	0.318 (0.047)
COD deficit (sec)	0.72 ± 0.15	0.74 ± 0.15	− 0.15 to 0.10	0.725	0.60 ± 0.11	0.72 ± 0.12	− 0.198 to − 0.017	0.433 (0.030)

*CMJ* countermovement jump, *RSI* reactive strength index, *DJ* drop jump, *V*_*IFT*_ final velocity at 30–15IFT, *COD* change-of-direction, *SSG* small sided games, *HIIT* high intensity interval training, *ANCOVA* analysis of covariance, *CI* confidence interval, $\eta_{p}^{2}$ partial eta squared

Significant differences between SSG and HIIT groups were found in the baseline (pre-intervention) for the CMJ (*p* = 0.014), CMJ free arms (*p* = 0.026), Single Hop Right (*p* = 0.030), Triple Hop Left (*p* = 0.015), Crossover Hop Right (*p* = 0.013), RSI DJ 20 cm (*p* = 0.045). No significant differences between groups were found at baseline for Single Hop Left (*p* = 0.127), Triple Hop Right (*p* = 0.068), Crossover Hop Left (*p* = 0.263), Single Hop Symmetry Angle (*p* = 0.874), Triple Hop Symmetry Angle (*p* = 0.830), Crossover Hop Symmetry Angle (*p* = 0.331), RSI DJ 30 cm (*p* = 0.066) and RSI DJ 40 cm (*p* = 0.051).

No significant differences between SSG and HIIT groups were found in the post-intervention in ANCOVA for the CMJ (*p* = 0.271), CMJ free arms (*p* = 0.453), Single Hop Right (*p* = 0.750), Single Hop Left (*p* = 0.318), Triple Hop Right (*p* = 0.263), Triple Hop Left (*p* = 0.346), Crossover Hop Right (*p* = 0.589), Crossover Hop Left (*p* = 0.130), Single Hop Symmetry Angle (*p* = 0.598), Triple Hop Symmetry Angle (*p* = 0.671), Crossover Hop Symmetry Angle (*p* = 0.938), RSI DJ 20 cm (*p* = 0.802), RSI DJ 30 cm (*p* = 0.433), RSI DJ 40 cm (*p* = 0.880).

Descriptive statistics of pre- and post-test sprinting, COD and aerobic fitness status values, within-group and between-group analysis can be found in Table 4. No significant differences between pre-post testing were found in the SSG group for the case of 10-m sprinting (*p* = 0.672) and 30-m sprinting (*p* = 0.341). Significant differences in both SSG and HIIT groups for V_IFT_ (*p* < 0.05)_,_ COD time (*p* < 0.05) and COD deficit (*p* < 0.05). Furthermore, significant differences were found at 10-m sprinting (*p* = 0.022) and 30-m sprinting (*p* = 0.027) for HIIT group.

Significant differences between SSG and HIIT groups were found in the baseline (pre-intervention) for the 10-m sprinting (*p* < 0.001), 30-m sprinting (*p* = 0.002), and COD time (*p* = 0.002). No significant differences were found between groups at baseline for the variables of COD deficit (*p* = 0.726), and V_IFT_ (*p* = 0.589). No significant differences between SSG and HIIT groups were found in ANCOVA in the post-intervention for the 10-m sprinting (*p* = 0.271), 30-m sprinting (*p* = 0.43), V_IFT_ (*p* = 0.750), COD time (*p* = 0.318), and COD deficit (*p* = 0.433).

**Fig. 1:**
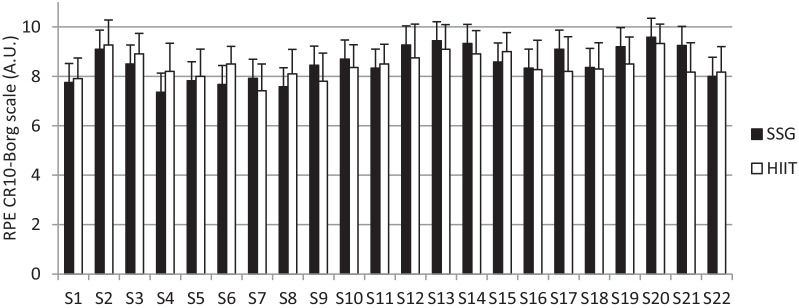
Rating of perceived exertion (RPE) reported by players over the training sessions (S) in the small-sided games (SSG) and running-based high-intensity interval training (HIIT)

The Fig. 1 presents the reported average RPE scores across the training sessions for both groups. No significant differences (*p* = 0.702) were found between the average RPE in the total of sessions between SSG (8.5 ± 0.3 A.U.) and HIIT (8.4 ± 0.4 A.U.).

## Discussion

The aim of this study was to compare the effects of small-sided games (SSGs) and running-based high-intensity interval training (HIIT) on the body composition and physical fitness of youth female soccer players. Our results showed no significant between-group differences after training intervention for all anthropometry measures, acceleration, aerobic fitness status or overall jump measures. Additionally, no significant between-group differences were revealed for the accumulated training intensity during the training interventions.

Overall, both SSGs and HIIT interventions revealed effective positive responses regarding the anthropometry measures, jump, sprint, COD, and locomotor performance. Indeed, other studies showed the effectiveness of both training methods in the short term, which is in line with the results of the present study [39, 40]. Regarding the jump (vertical and horizontal) performance category, a recent meta-analysis that compared the effects of both SSGs and HIIT interventions on the physical performance of soccer players concluded that there are no significant differences between the two training methods for vertical or horizontal jump performance [41]. Prescribing exercises that stimulate positive adaptations in jump performance is of paramount importance in team sports such as soccer, as it seems to be correlated with kicking ball velocity [42] and is considered a determinant factor of the final outcome of a soccer match [43]. Although both SSGs and HIIT produced positive responses to jump performance in our study, there are other methods that are more effective in producing adaptations in jump performance, such as strength training [44, 45].

Considering the acceleration (10 m) and sprinting (30 m) performance, accelerations showed no significant differences between the SSG and HIIT groups, while for sprint performance, only the HIIT group showed significant improvements. These results were expected, as during SSGs, there are many accelerations and decelerations mainly due to the smaller pitch sizes and other drill constraints [21, 46]. Therefore, this type of training method can produce similar acceleration responses as more controlled drills, such as HIIT drills, which is consistent with other studies [39, 47]. However, one of the biggest threats of using SSGs as a standalone method of soccer training is that some physical determinants of the game, such as linear sprinting over greater distances (e.g., 30-m sprints), may not be accomplished during SSGs [48]. That is, for producing better adaptations in 30-m sprint performance, athletes would benefit from running-based short-interval HIIT exposures [19].

Meanwhile, the SSG group presented greater adaptations to COD ability measures than the HIIT group after the eight-week training intervention. In a recent systematic review, it was concluded that the volume of CODs and their respective angles during a training session were determinants for adaptations to occur [49]. Thus, when prescribing HIIT drills in a linear fashion, it is not expected that this type of training will produce better improvements than SSGs. Indeed, a study conducted on 33 young soccer players revealed that a six-week SSG training intervention improved COD performance by 7.23%, while no significant differences were found for the 30-m sprint test, which aligns with the results of the present study [50]. Another systematic review with meta-analysis revealed that SSGs are more effective than HIIT training for developing COD ability, representing moderate to large improvements associated with SSG training [51]. For those reasons, it seems reasonable to prefer SSGs over linear HIIT tasks for COD development, as used in the present study, or at least to add two or more COD at different angles during HIIT drills.

As revealed in the present study, previous studies have demonstrated that SSG and HIIT training interventions are capable of producing similar locomotor positive responses [40, 52]. Indeed, some studies revealed improvements in VO2max of approximately 7–11% using HIIT interventions [39, 53]. Other studies exploring the effects of SSGs on aerobic performance showed similar improvements (7%), which is in line with our findings regarding aerobic responses after these two methods of training interventions. Although the majority of the above-mentioned studies were conducted on young and adult male soccer players, it seems that SSG and HIIT interventions are effective training methods for developing the aerobic performance of female soccer players.

The lack of differences in SSG and HIIT training interventions’ effects on RPE values in the present study is in line with a study conducted on 16 youth soccer players, which revealed that both training methods induced similar physiological responses in terms of training intensity [54]. However, in that study, the authors only used a 4v4 SSG format for one week, while in the present study, we used 2v2 and 3v3 formats for eight weeks, which can make this comparison complicated. However, although no significant between-group differences were revealed in the present study, it was observable that in some sessions, the RPE values were higher in the HIIT group than in the SSG group. This is in contrast with other studies that showed that the SSG groups had lower RPE values than HIIT groups, mainly associated with the enjoyment characteristics of SSGs [54, 55]. The greater RPE values associated with the SSG group in the present study may be a consequence of the difficulty of controlling the drill intensities during SSGs [17].

This study had some limitations. One of the main limitations was that the players did not train during the season before the beginning of the present study due to COVID-19 pandemic restrictions. Such a limitation could have biased the pre-post data, as the adaptations revealed could have also been influenced by the lack of training during the antecedent season. Future studies could use external training intensity measures to enrich knowledge regarding the demands of both training interventions in female soccer players.

## Conclusion

This study revealed that after only eight weeks of both SSG and HIIT training interventions, female athletes produced similar responses regarding anthropometry, acceleration, and locomotor performance. However, the HIIT training is more suitable for producing greater adaptations in long linear sprint capacity. Conversely, SSGs were more effective for producing adaptations in the time taken to complete COD tasks. Moreover, the accumulated perceived training intensities were similar for both groups after the eight-week training intervention. Coaches working with female athletes should be aware of differences in responses when using HIIT or SSGs. Combining SSGs with a conscient volume of HIIT can be more beneficial than using only one training method in female soccer players.

## Data Availability

The datasets generated and analyzed during the current study are not publicly available due to ethical restrictions, however, they are available from Sinan Nayıroğlu on reasonable request.
